# Effects of exercise and diet change on cognition function and synaptic plasticity in high fat diet induced obese rats

**DOI:** 10.1186/1476-511X-12-144

**Published:** 2013-10-08

**Authors:** Jinhee Woo, Ki Ok Shin, So Young Park, Ki Soeng Jang, Sunghwun Kang

**Affiliations:** 1 Department of Physical Education, Laboratory of Exercise Physiology, College of Sports Science, Dong-A University, 840 Hadan2-dong, Saha-gu, Busan, Korea; 2 Departments of Pharmacology, Medical Sciences Research Institute, College of Medicine, Dong-A University, Busan, Korea

**Keywords:** Exercise, Cognition function, Synaptic plasticity, High fat diet, Memory span

## Abstract

**Background:**

Nutritional imbalance-induced obesity causes a variety of diseases and in particular is an important cause of cognitive function decline. This study was performed on Sprague Dawley (SD) rats with 13-weeks of high fat diet-induced obesity in connection to the effects of regular exercise and dietary control for 8 weeks on the synaptic plasticity and cognitive abilities of brain.

**Methods:**

Four weeks-old SD rats were adopted classified into normal-normal diet-sedentary (NNS, n = 8), obesity-high fat diet-sedentary (OHS, n = 8), obesity-high fat diet-training (OHT, n = 8), obesity-normal diet-sedentary (ONS, n = 8) and obesity- normal diet-training (ONT, n = 8). The exercise program consisted of a treadmill exercise administered at a speed of 8 m/min for 1–4 weeks, and 14 m/min for 5–8 weeks. The Western blot method was used to measure the expression of NGF, BDNF, p38MAPK and p-p38MAPK proteins in hippocampus of the brain, and expressions of NGF, BDNF, TrkA, TrkB, CREB and synapsin1 mRNA were analyzed through qRT-PCR.

**Results:**

The results suggest cognitive function-related protein levels and mRNA expression to be significantly decreased in the hippocampus of obese rats, and synaptic plasticity as well as cognitive function signaling sub-pathway factors were also significantly decreased. In addition, 8-weeks exercises and treatment by dietary change had induced significant increase of cognitive function-related protein levels and mRNA expression as well as synaptic plasticity and cognitive function signaling sub-pathway factors in obese rats. In particular, the combined treatment had presented even more positive effect.

**Conclusions:**

Therefore, it was determined that the high fat diet-induced obesity decreases plasticity and cognitive function of the brain, but was identified as being improved by exercises and dietary changes. In particular, it is considered that regular exercise has positive effects on memory span and learning capacity unlike dietary control.

## Introduction

Obesity-induced oxidative stress not only causes inflammatory reactions resulting in abnormalities of the immune system [1] but also transforms functions of protein, lipid and DNA, triggering neurodegenerative diseases such as Alzheimer’s disease and Parkinson’s disease [2,3] as well as aging of brain function, cognitive impairment [4], and is reported to affect apoptosis of neuronal cells. As aforementioned, the obesity is predicted to affect cognitive functions including reduced learning capacity and memory impairment, however, the mechanism of which brain function it affects remains unclear. Cognitive impairment includes reduced concentration, poor progress in learning, memory impairment where its molecular mechanism is manifested through change of hippocampal function in the brain. Furthermore obesity has been reported to cause problems in cAMP and protein kinase A dependent synaptic plasticity [5]. Thus, it is thought that identification of the relationship between obesity and synaptic plasticity will help in understanding the mechanism between obesity and cognitive function.

Regular exercise not only prevents and treats obesity-induced metabolic imbalance but also prevents brain damage. It also enhances learning and memory span along with stimulation of neurogenesis [6]. Even more surprising is the fact that exercise reinforces the capacity for functional recovery from brain damage and is attributed to prolonging the process of aging effects on brain function [7,8]. Exercise-induced increases of nerve growth factor (NGF) and brain-drived neurotrophic factor (BDNF) in the hippocampus have been known to reinforce cognitive function [9,10], however the question still remains as to whether exercise-induced reinforcement of learning and memory span is due to increases of hippocampal NGF and BDNF or not.

NGF and BDNF, also known as neurotransmitters, are drawing attentions as major mediators of cerebral synaptic plasticity, while BDNF is known to be involved in controlling food intake and glucose homeostasis [11]. In the case of genetically obese animals, it was reported that TrkB, a BDNF receptor was depressed [12], and that high fat / high sugar diet reduced expression of hippocampal BDNF, resulting in deterioration of learning as well as memory span [13]. NGF and its receptor TrkA are also known to be suppressed in connection to disease and nerve damage [14], and were identified as having negative effects on learning and memory as a result of high-intensity acute exercise [15]. However, it was reported that norepinephrine increased as a result of regular exercise facilitated by neurocyte regeneration so as to increase BDNF expression of cultivated hippocampal cells [16]. Additionally a study on mice reported that the expression of BDNF mRNA increased after a three day running exercise routine [17]. From these reports, it could be considered that regular exercise increases expressions of NGF and BDNF [10]. Therefore, the studies that had observed changes of synaptic plasticity such as NGF, BDNF, p38MAPK, cAMP response element-binding protein (CREB) and synapsin 1 in rats with obesity triggered by the energy imbalance from high fat diet, are still scarce, and in particular, there is no study to this date implementing the use of regular exercise and dietary change in these obese models. Therefore, this study aimed to define the relationship between obesity and neurogenesis in rats with acquired obesity caused by high fat diet, and to identify the effects of regular aerobic exercise and dietary change on synaptic plasticity markers and cognitive function.

## Methods

### Animals

The adopted subjects of this study were 40 heads of four week old white male Sprague–Dawley (SD) rats. Out of 40 heads, 32 were induced obesity through 13-weeks of a high fat diet while the other eight heads were fed with a normal diet. After 13 weeks, all study rats were classified into five groups which were the normal-normal diet-sedentary group (NNS), obesity-high fat diet-sedentary group (OHS, n = 8), obesity-high fat diet-training group (OHT, n = 8), obesity-normal diet sedentary group (ONS, n = 8) and obesity-normal diet-training group (ONT). These five groups were bred for eight weeks.

Obesity was induced in the high fat diet groups as provided by 25 g of feeds containing all essential nutrients, vitamins, minerals and 45% of fat per one head daily with water, while the normal diet groups were provided 25 g of the ordinary feeds per one head daily with water. During the study duration, the dietary intakes were measured at the same time (19:00) each day, and the weights were measured at a certain time for once a week by using the computing scale. In order to prevent temporary weight change due to dietary intake, these measurements were performed after removing the feeding container 2 hours before. The study was carried out in accordance with the Guidelines laid down by the NIH in the US regarding the care and use of animals for experimental procedures and was approved by DONG-A University Institutional Animal Care and Use Committee.

### Exercise program

Treadmill running was performed as the exercise type. For the exercise protocol, during the period of weeks 1 ~ 4, exercise was performed five days per week, of 40 minutes duration and during the period of weeks 4 ~ 8, exercise was performed five days per week, for a duration 60 minutes per day. During weeks 1 ~ 4, treadmill exercise was conducted where the slope was fixed to zero degrees, and a 5 minute – warm up at a speed of 2 m/min, followed by 30 minute main exercise bout at a speed of 8 m/min concluded by five minute final cool down session at a speed of 5 m/min. For weeks 5 ~ 8, a 10 minute – warm up at a speed of 8 m/min, a 40 minute- main exercise bout was at a speed of 14 m/min with a 10 minute-final cool down at a speed of 11 m/min [18].

### Cognitive function test

A Morris water maze (MWM) was employed in order to verify the effectiveness of the brain’s cognitive function [13]. The study equipment was a circular swimming pool (diameter 150 ~ 180 cm, height 50 cm) filled with water at a depth of 25 ~ 30 cm, 25°C, to which skim milk was mixed to make it opaque in order to prevent the platform installed from being seen. The platform was made with acrylic material of a diameter of 10 cm and height of 23 ~ 24 cm was installed in the pool to be invisible above the water while the spatial cues were provided on the surrounding walls for animals to learn the location of the platform. The water (22 ~ 24°C) was made opaque with white nontoxic biodegradable dye to prevent the rats form seeing the platform. The experiment animals were trained on the water maze using 2 consecutive trials per day for 3 days and placed into the bathtub the wall from one of the equally spaced start locations that were randomly changed every trial. Each trial lasted until the rat had found the platform or for a max 1 min [18].

### Tissue sample

For the timing of tissue sampling in this study, they were conducted at 48 hours after the completion of training for the training groups in order to rule out temporary exercise effects from treadmill training. For such samplings, the feed supply was discontinued 12 hours prior to the samplings, however drinking water was continually supplied. The study animals, after arrival at the laboratory, had taken sufficient rest and they were put into anesthesia by using ethyl ether, after skulls were removed to collect brain hippocampus (5 g) samples. The tissue samples were stored in a deep freezer (NIHOW freezer, Japan) at −70°C until analysis.

### Analysis content

#### Western blot

To extract protein from the hippocampus, the tissues were crushed after adding a solution containing 150 mM Nacl, 5 mM EDTA, 50 mM Tri-HCI (pH8.0), 1%-NP 40, 1 mM aprotinin, 0.1 mM leupeptin, and 1 mM pepstatin and then the solution was put into centrifugation for 30 minutes at 14,000 rpm. Supernatants were collected and assayed for protein content prior to storage at −70°C. Protein sample were mixed Laemmli Sample Buffer (LSB) and placed in a boiling water bath for 5 min. Proteins were resolved by 10, 12 or 15% SDS-polyacrylamide gel electrophoresis (SDS-PAGE; each loaded with same μg of total protein per lane), and transferred to nitrocellulose membranes. Then, the membrane was blocked in the solution of Phosphate - buffered saline (PBS) (Nacl 8 g, KCI 0.2 g, Na2HPO4 1.44 g, KH2PO4 0.24 g, PH 7.4) added with 5% skim milk. Thereafter, the samples were reacted in a solution mixed with phospho antibody NGF (1:1000; Santa Cruz Biotechnology), BDNF (1:1000; Santa Cruz Biotechnology), phospho antibody P38 (1:1000; Santa Cruz Biotechnology) and phospho antibody for one hour, and washed for 1×15 min and 2×5 min in a PBS solution containing 0.1% 0.1% tween - 20. Each membrane was reacted for one hour in a solution added with goat anti mouse and rabbit 1gG conjugated secondary antibody respectively, and was measured for immune-reactive bands through a Kodak film. The relative strengths of bands were quantitated by a densitometry (Sci - Scan, UUSB).

#### Quantitative real time PCR

Total RNA was separated by using the RNA STAT-60 kit (TEL-TEST, Inc., Friendswood, TX, USA) according to the protocol, and the absorbance was measured at 260 nm in addition to analyses of NGF, BDNF, TrkA, TrkB, MAPK1, CREB and Synapsin1 i.e., total RNA of 100 ng was converted to cDNA in use of TaqMan EW RT-PCR core reagent (Perkin-Elmer, Branchburg, NJ, USA), with subsequent designing of primers sequences of the former and the latter by Integrated DNA Technologies (Coralville, IA, USA) as follows.

NGF - forward(5′-TTCCAGGCCCATGGTACAA-3′), reverse(5′-GGTGGATGAGCGCTTGCT-3′)

TrkA – forward(5′-GCCGCCCTCTTCCTTTCT-3′), reverse(5′-ATTTGCTCCTCTGTCCACATTTG-3′)

BDNF – forward(5′-GCGCCCATGAAAGAAGCA-3′), reverse(5′-CACAGCTGGGTAGGCCAAGT-3′)

TrkB – forward(5′-TTTCCGCCACCTTGACTTG-3′), reverse(5′-ACAGGAACACGTGAACGGATT-3′)

CREB – forward(5′-CAGTGCCAACCCCGATTTA-3′), reverse(5′-TTGCTCCTCCCTGGGTAATG-3′)

Synapsin1 – forward(5′-GCCTTCAGCATGGCACGTA-3′), reverse(5′-CAGCATACTGCAGCCCAATG-3′)

MAPK1 – forward(5′-ATTTGGTCTGTGGGCTGCAT-3′), reverse(5′-TCCTGGGAAGATAGGCCTGTT-3′)

For RT reaction phase, cultivation was done for the initial two minutes at 50°C for uracil glycosylase (UNG) activation, followed by reverse transcription at 60°C for 30 minutes. The completion phase for UNG inactivation was performed at 95°C for 5 minutes, and thereafter, a 40 cycle was carried out at 94°C for 20 followed by for 1 minute at 62°C.

### Statistical analysis

Data were analyzed using SPSS Software Version 18.0 for Window (SPSS Inc, Chicago, IL) and were presented as mean ± SE unless otherwise indicated. The change in weight induced by the diet method was analyzed through a two-way repeated measures ANOVA, and paired t-tests were performed for time-time interactions, and for any intergroup difference observed, one-way ANOVA was performed in addition to Duncan’s Post-hoc analysis. An analysis of variance (ANOVA) with 2-way repeated measures was conducted for analyzing data of the water maze. For the results of the cognitive function and for the expressions of hippocampal neurotrophins as well as synaptic plasticity gene, one-way ANOVA was in addition to Duncan’s Post-hoc analysis. Statistical significance was declared when p values were less than 5% level.

## Results

**Figure 1:**
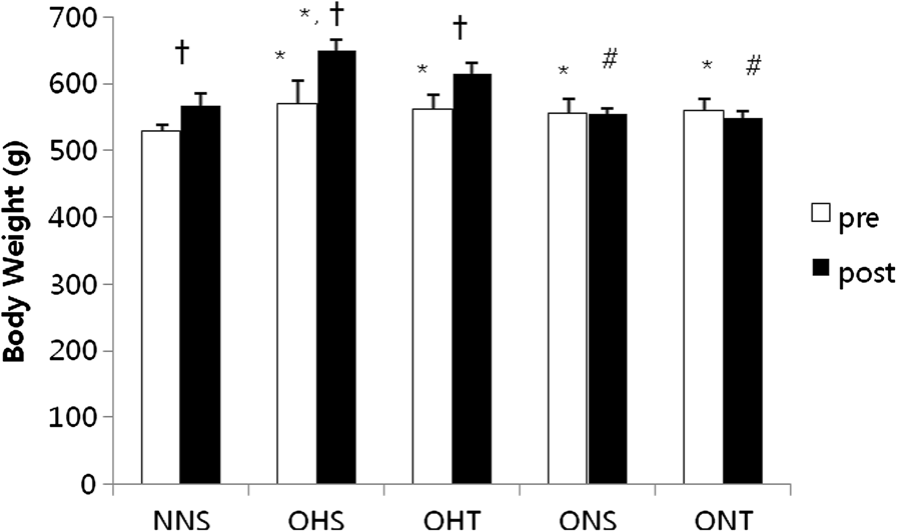
**Comparison of body weight of high fat diet induced-obesity after 8-weeks treatment.** Mean ± SE, *p < 0.05 vs NNS, #p < 0.05 vs OHS, ^†^p < 0.05 pre and post. NNS; normal-normal diet-sedenstary, OHS; obesity-high fat diet-sedentary, OHT; obesity-high fat diet-tarining, ONS; obesity-normal diet-sedentary, ONT; obesity-normal diet-training.

Figure 1 presents the comparison of weights before and after 8-weeks treatment in rats with high fat diet-induced obesity. Compared to the weights before treatment, there were significant increases of NNS, OHS and OHT after treatment, however ONS and ONT did not shown differences. ONS and ONT presented significantly lower post-treatment intergroup variation compared to post-treatment OHS (p < 0.05).

**Figure 2:**
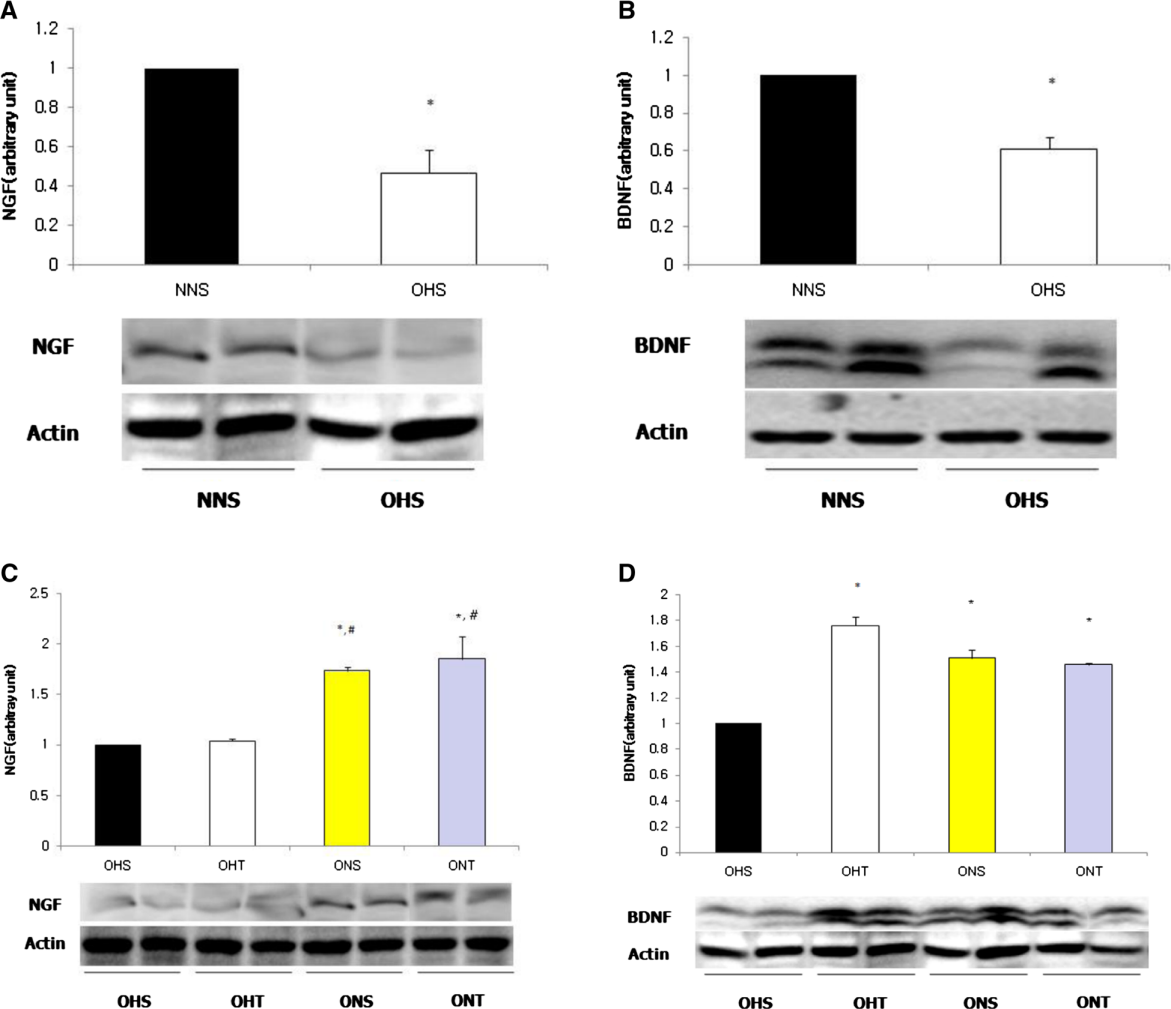
**Comparison on protein expressions of NGF (A) and BDNF (B) in hippocampus of rats with high fat diet-induced obesity.** Comparison on protein expressions of NGF **(C)** and BDNF **(D)** after 8-weeks treatment (Exercise, normal diet, exercise and normal diet). Values are means ± SE. *p < 0.05 vs NNS **(A, B)** or OHS **(C, D)**, #p < 0.05 vs OHT.

OHS displayed significantly lower expressions of hippocampal NGF and BDNF protein compared to NNS (p < 0.05). However, For BDNF after 8-weeks treatment, OHT, ONS and ONT had presented significantly higher levels than OHS (p < 0.05), and for NGF, the levels of ONS and ONT were significantly higher than OHS and OHT (p < 0.05) (Figure 2).

**Figure 3:**
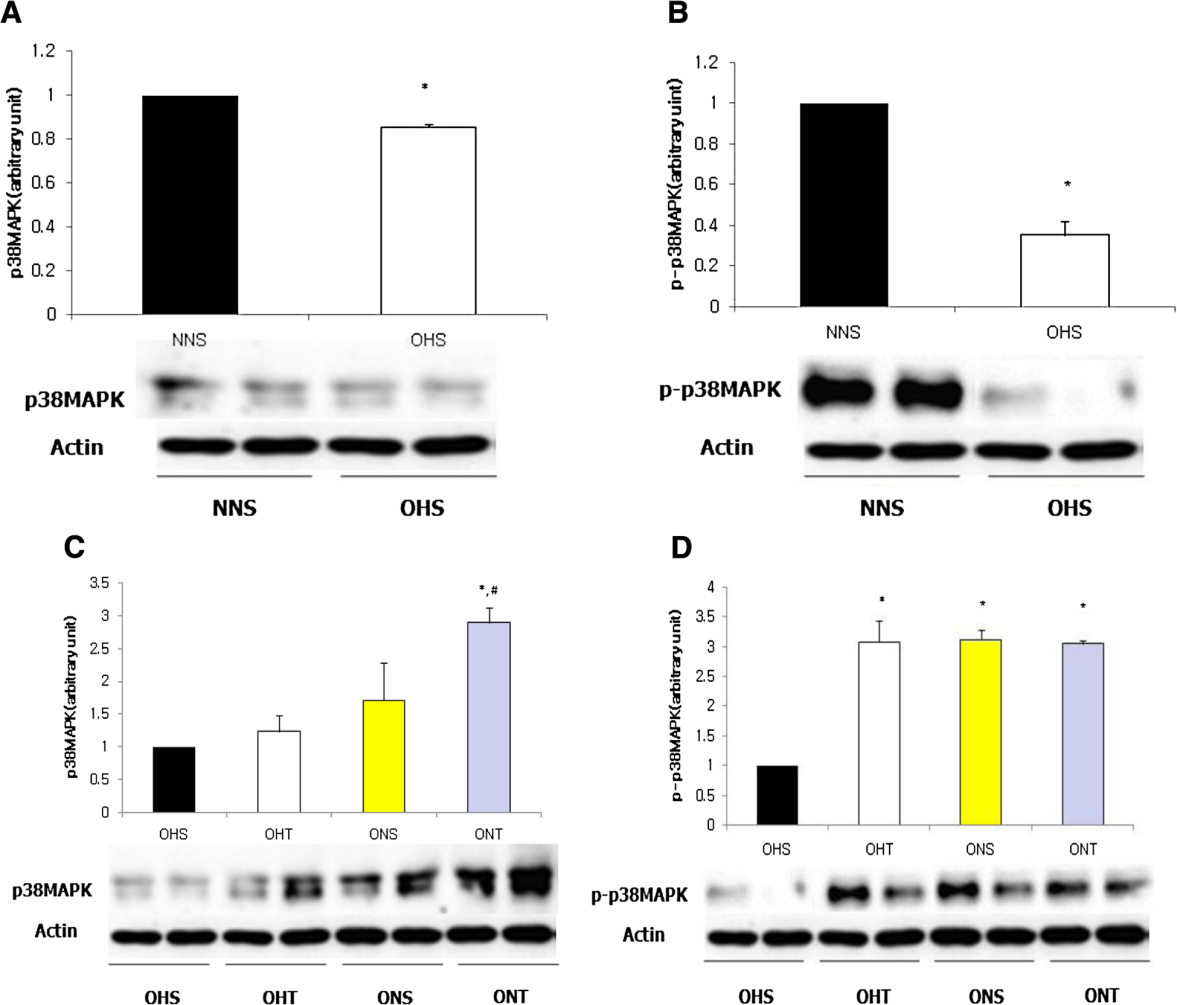
**Comparison on protein expressions of p38MAPK (A) and p-p38MAPK (B) in hippocampus of rats with high fat diet-induced obesity.** Comparison on protein expression of p38MAPK **(C)** and p-p38MAPK **(D)** after 8-weeks treatment (Exercise, normal diet, exercise and normal diet). Values are means ± SE. *p < 0.05 vs NNS **(A, B)** or OHS **(C, D)**, #p < 0.05 vs OHT.

For protein expressions of p38MAPK and p-p38MAPK, the OHS group had shown significantly lower levels than NNS (p < 0.05). However, after 8-weeks of treatment, p38MAPK displayed the highest level in the ONT group (p < 0.05), and p-p38MAPK presented significantly higher levels in the OHT group, ONS group and the ONT group compared to the OHS group (p < 0.05) (Figure 3).

**Figure 4:**
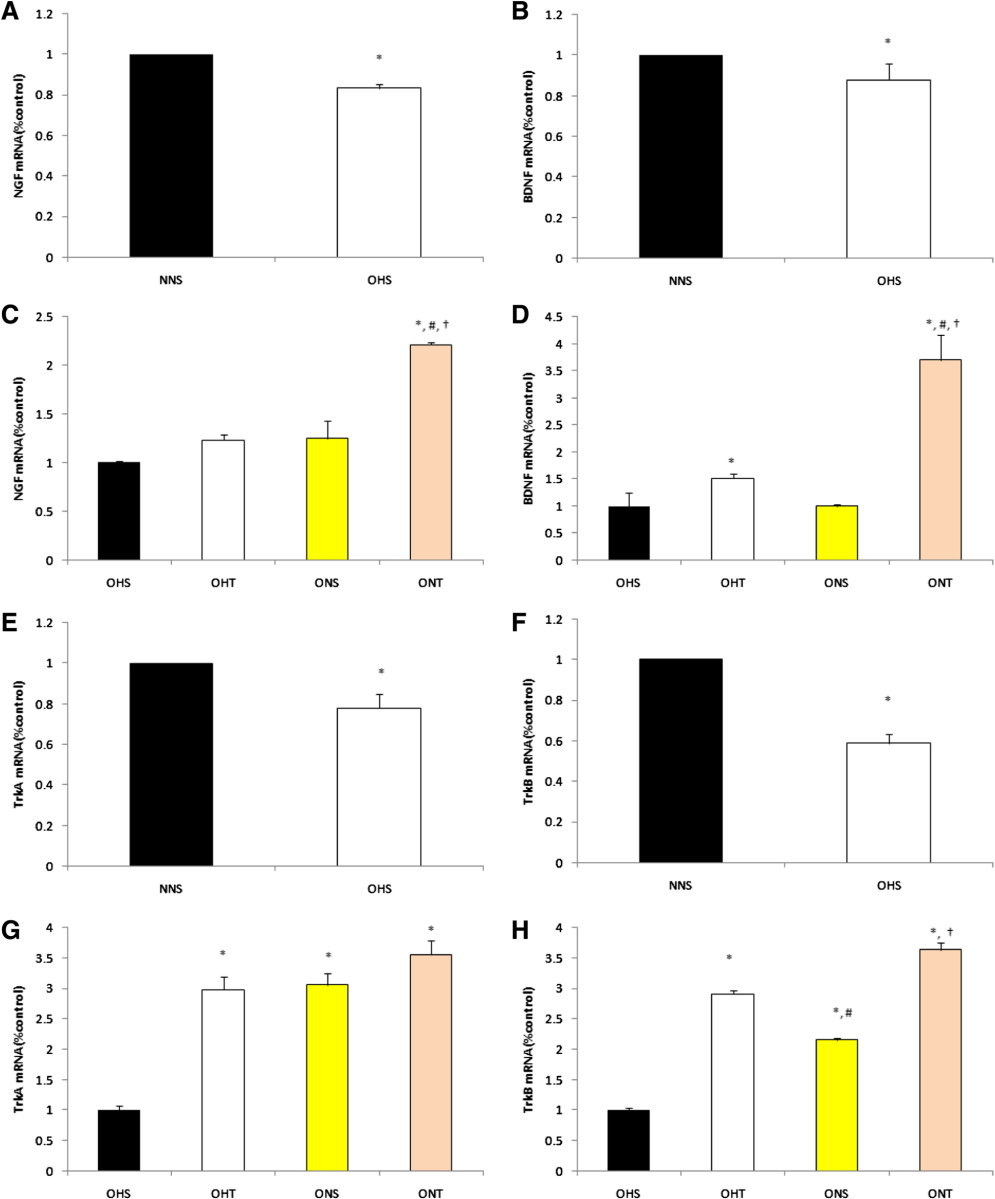
**Comparison on mRNA levels of NGF (A), BDNF (B), TrkA (E) and TrkB (F) in hippocampus of rats with high fat diet-induced obesity.** Comparison on mRNA levels of NGF **(C)**, BDNF **(D)**, TrkA **(G)** and TrkB **(H)** after 8-weeks treatment (Exercise, normal diet, exercise and normal diet). Values are means ± SE. *p < 0.05 vs NNS **(A, B, E, F)** or OHS **(C, D, G, H)**, #p < 0.05 vs OHT, †p < 0.05 vs ONS.

As shown in Figure 4, BDNF mRNA significantly decreased in rats with high fat diet-induced obesity (p < 0.05), and TrkB significantly decreased as well (p < 0.05). However, it was identified that all of NGF, BDNF, TrkA and TrkB mRNA were significantly increased through 8-weeks exercise and dietary change (p < 0.05).

**Figure 5:**
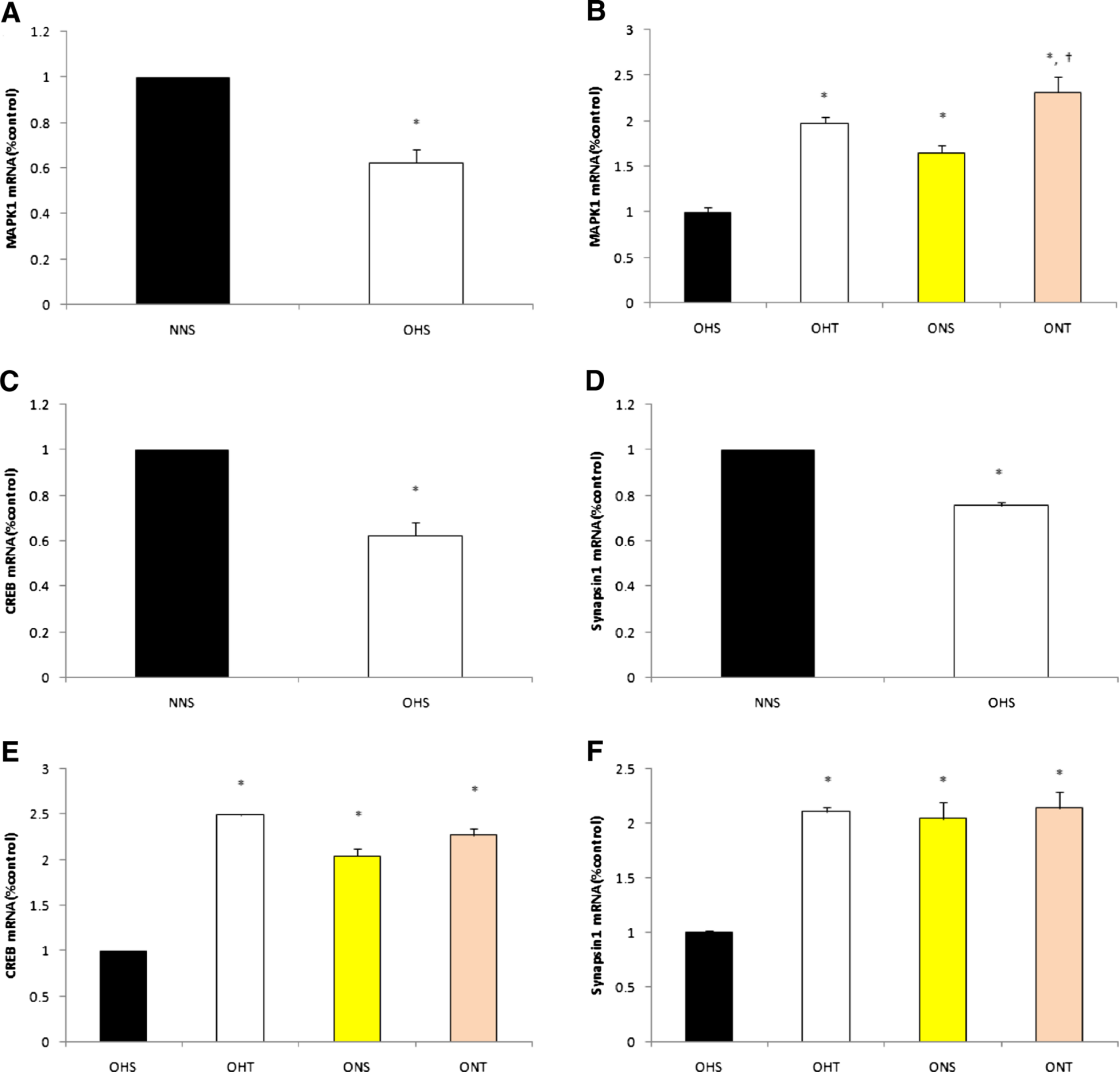
**Comparison on mRNA levels of MAPK1 (A), CREB (C) and Synapsin1 (D) in hippocampus of rats with high fat diet-induced obesity.** Comparison on mRNA levels of MAPK1 **(B)**, CREB **(E)** and Synapsin1 **(F)** after 8-weeks treatment (Exercise, normal diet, exercise and normal diet). Values are means ± SE. *p < 0.05 vs NNS **(A, C, D)** or OHS **(B, E, F)**.

As shown in Figure 5, NGF and BDNF mRNA presented the highest levels in the ONT group that had 8-weeks combined diet control and training (p < 0.05). TrkA mRNA displayed high levels in the OHT, ONS, and the ONT group than the OHS group after treatment while TrkB mRNA and MAPK1 mRNA presented the highest levels in the ONT group (p < 0.05). Additionally, post-treatment levels of CREB mRNAand Synapsin1 mRNA were higher in the OHT, ONS and ONT groups compared to the OHS group (p < 0.05).

**Figure 6:**
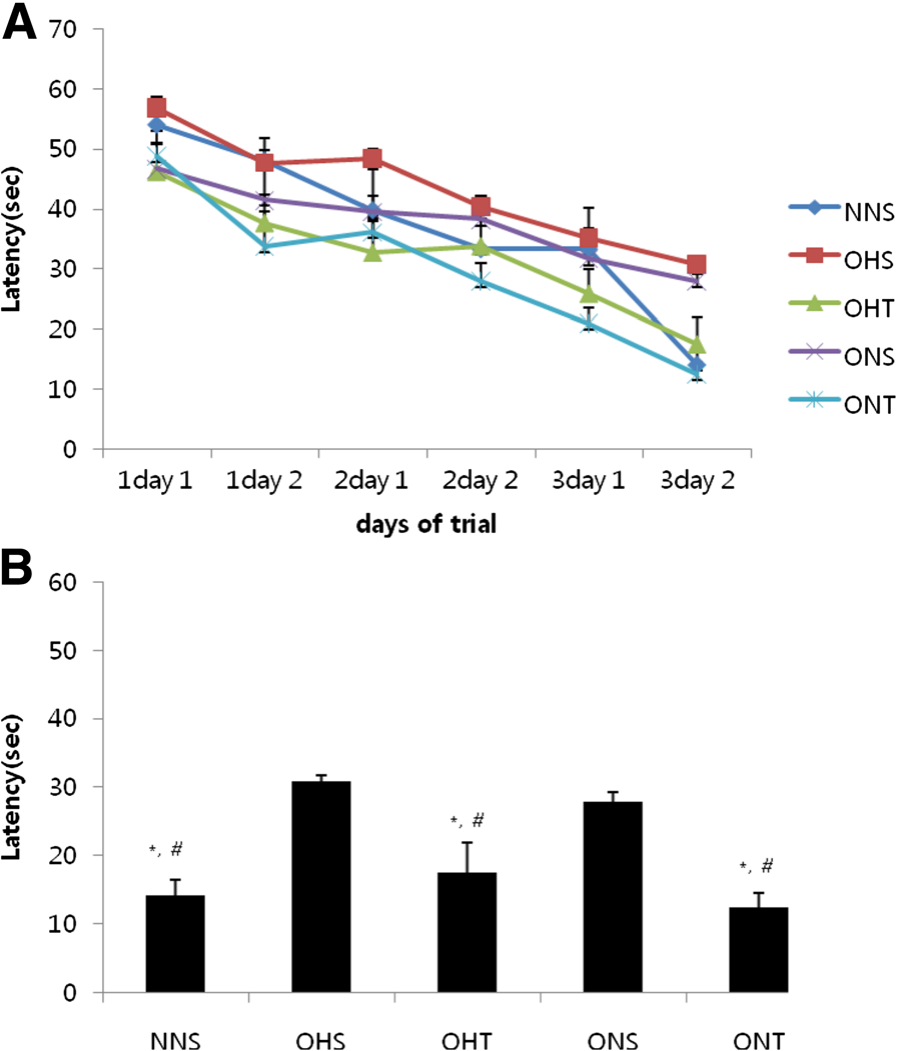
**Effects of diet and exercise on spatial learning performance using the water maze. (A)** Effects of between group and time an interaction was showed. In addition, there were significant differences in the group and time respectively. **(B)** In the final period the 3 days NNS, OHS and ONT compared to the OHS and ONS significantly lower. Values are means ± SE. *p < 0.05 vs NNS, #p < 0.05 vs ONS.

As shown in Figure 6, spatial learning test six times a significant interaction effect was found between group and time. In addition, there were significant differences between group and time respectively (A). As shown in Figure 6B, the sixth time in the OHS group higher compared to the NNS, OHT and ONT significantly (p < 0.05). Also, in the ONS group higher compared to the NNS, OHT and ONT significantly (p < 0.05). We found that spatial learning reduces time to exercise.

## Discussion

This study was conducted by establishing a hypothesis that regular exercise and dietary change will have positive effects on the brain’s plasticity and cognitive function as a result of high fat induced obesity in rats. Induction of obesity by high fat diet was identified as having negative effects (inactivity) on all protein factors related to cognitive function. However, exercise and dietary change resulted in improvement of cognitive function protein factor and the level of mRNA (activity). In particular, regular exercise had positive effects on activation of sub signaling pathways as well as on improvement in cognitive ability tests.

According to a recent study, it was reported that proper stress increased exercise-performing ability in young animals, while it improved learning ability in aged animals [19]. Particularly, stress such as regular exercise had been reported to prolong the deterioration of cognitive function in connection to aging [20], and to reduce risks of various neuropathies [21,22]. It was considered that regular exercise activated metabolic actions so as to trigger continuous gene alterations. The environmental impact such as exercise on cognitive function, in particular, causes BDNF gene alteration [23,24], and has positive effects on reduction of apoptosis by improvements of NGF and TrkA [25]. In addition, it was suggested that regular exercise improved the hippocampal levels of NGF and TrkA as well as BDNF and TrkB so that it was effective for learning and cognitive abilities [9,10]. Nevertheless, BDNF and NGF are understood to be reduced or overly expressed in the state of disease [14], and stress such as high-intensity acute exercise is reported to cause damages to learning and memory [15].

From the results of this study, it was identified that the rats with high fat diet-induced obesity had significantly lower levels of cognitive function-related protein expression (NGF, BDNF, p38MAPK). This result confirmed the findings of previous studies that obesity could have negative effects on NGF, BDNF and p38MAPK. However due to regular 8-weeks treadmill exercise and dietary change, the protein levels of BDNF and NGF had increased significantly, and the activity of p38MAPK displayed high levels, indicating exercise and dietary control had effects on weight loss and improved cognitive abilities. Meanwhile, with high fat diet-induced obesity, expressions of NGF and TrkA mRNA as well as BDNF and TrkB mRNA were presented significantly low. As suggested by previous studies, obesity and diseases reduces the expressions of NGF [14] and BDNF [26], and it is considered that high fat diet-induced obesity affects hippocampal cognition-related factors. However, these results were consistent with the results of previous studies [9,23] that this study had identified regular exercise and dietary change had resulted in significant increase of NGF and TrkA as well as BDNF and TrkB mRNA.

In general, NGF signaling is composed by activation of the 2-folds delivery system [27]. First, it is spread through continuous activation of mitogen activated protein kinases (MAPK) by Erk phosphorylation, and secondly it is spread by activation of PI3K/Akt signaling pathway as reported by Williams et al., [28]. According to a recent study, it was identified that regular exercise improves the protein levels of Akt, CREB and BDNF [29], and activates MAPK that improves synaptic activity [10]. These findings report that regular exercise had effects not only on CREB phosphorylation but also on increase of MAPK/Erk phosphorylationin the hippocampus of rat [30], particularly, in cases of diabetes-induced rat, it activates phosphorylation of Erk and CREB, and increases the level of NGF so as it induces activations of MAPK/Erk1/2 signaling pathway and CREB in the hippocampus even more [10]. The main results of reported by those previous studies proves that regular exercise is an important element and essential for expression of brain function-related signal transmitting protein. In general, it has been known that BDNF decline causes excessive eating and develops obesity by inactivation of sub pathways [31], and high fat diet reduces the levels of synaptic plasticity and cognitive function, bringing negative effects on learning and memory span [13]. As suggested previously, regular exercise facilitates synthesis of MAPK, CREB and synapsin 1 which are sub pathways owning to activations of BDNF and NGF [32] and activates the phosphorylation process [33] that is likely to have effects on synaptic plasticity and cognitive abilities. That means, regular exercise enables manifestation of extracellular activations of NGF and BDNF, which induces intracellular activations of CREB and Synapsin 1 in MAPK and the nucleus. Such mechanisms are known to have effects on emission of neurotransmitters, maintenance of synaptic connection and extension of neuritis, resulting in improvement of learning and memory span as well as raising cognitive abilities as reported before [34,35]. Moreover, recent studies suggest that regular exercise improves hippocampal plasticity and learning via Akt, CREB and BDNF signaling [28], while the high levels of NGF and receptor TrkA improve learning and memory span [36].

Therefore, the imbalance of energy metabolism caused by high fat diet affects synaptic plasticity to be reduced and learning as well as cognition of the brain with low levels of NGF and BDNF, but with regular exercise and dietary change, it has been identified to be possible to improve the reduced synaptic plasticity and cognitive function. From the results of this study, it is indicated that the protein activations of BDNF, NGF and p38MAPK in the hippocampus of rats with high fat diet-induced obesity were inhibited resulting in significant reduction of such activations, and in addition, from the level of mRNA, the reduction tendency of TrkA, TrkB, MAPK1, CREB and Synapsin1 had been presented. Such results indicated that high fat containing diet intake causes reduction of signaling pathways related to cognitive function prolonging the time of Morris water maze (MWM) performance, as consistent with previous studies.

However, with regular treadmill exercises for 8 weeks and dietary change, the protein levels of BDNF and NGF were presented significantly high, together with high level of p38MAPK activation, verifying exercise and dietary change can improve cognitive abilities. In particular, the combined treatment was identified as even more effective. Additionally, the expression levels of mRNA in all of TrkA, TrkB, MAPK1, CREB and Synapsin1 were identified to be high from as a result of regular exercise, diet control and combined treatment. Given such results, regular exercise and diet control not only improve cognitive function-related proteins but also increase mRNA, in addition to induce activation of sub signaling pathway, resulting in improvement of synaptic plasticity as well as cognitive abilities of brain. Furthermore, compared to diet control, regular exercise presented effective in facilitating muscle memory by shortening MWM time owing to the improvement of even more cognitive function signaling pathways, so it thought that diet control and exercise could result in improvement of cognitive function only when they were co-performed.

## Conclusion

The high fat diet-induced obesity is considered to reduce plasticity and cognitive function of brain so as to reduce muscle memory, but exercise and dietary change is considered as having positive effects on synaptic plasticity and cognitive function sub pathway of brain. In particular, unlike the diet control, regular exercise activates neurogenesis so that it is considered as contributing to the muscle memory effect by presenting improvement of MWM ability that is directly associated with cognitive function.

## Abbreviations

SD: Sprague Dawley; NGF: Nerve growth factor; BDNF: Brain-drived neurotrophic factor; CREB: cAMP response element-binding protein; MAPK: Mitogen activated protein kinases; MWM: Morris water maze.

## Competing interests

The authors declare that they have no competing interests.

## Authors’ contributions

JW and SK designed the study, carried out the experiments, performed the statistical analysis. SYP carried out the experiments and KSJ collected the data. SK wrote the manuscript, and also participated in the execution and analysis of this study with KOS. All authors read and approved the final manuscript.
